# Association of vitamin D receptor genetic polymorphisms with the risk of infertility: a systematic review and meta-analysis

**DOI:** 10.1186/s12884-024-06590-0

**Published:** 2024-05-30

**Authors:** Asra Moradkhani, Mobin Azami, Srwa Assadi, Mobin Ghaderi, Asaad Azarnezhad, Yousef Moradi

**Affiliations:** 1 Student of the Research Committee, Kurdistan University of Medical Sciences, Sanandaj, Iran; 2 Cellular and Molecular Research Center, Research Institute for Health Development, Kurdistan University of Medical Sciences, Sanandaj, Iran; 3 Social Determinants of the Health Research Center, Research Institute for Health Development, Kurdistan University of Medical Sciences, Sanandaj, Iran; 4 Liver and Digestive Research Center, Research Institute for Health Development, Kurdistan University of Medical Sciences, Sanandaj, Iran

**Keywords:** Infertility, Miscarriage, Vitamin D receptor, Polymorphism, Meta-Analysis, Systematic review

## Abstract

**Background:**

The causes of infertility have remained an important challenge. The relationship between VDR gene polymorphisms and infertility has been reported, with controversial findings.

**Objective and rationale:**

We aimed to determine this relationship by conducting a systematic review and meta-analysis.

**Search methods:**

The study was started with the Preferred Reporting Items for Systematic Reviews and Meta-Analyses (PRISMA) declaration and the final draft was registered as a protocol in PROSPERO (ID: CRD42023416535). The international electronic databases including PubMed (Medline), Scopus, Web of Sciences, and Cumulative Index to Nursing and Allied Health Literature (CINHAL) were searched until January 30, 2023, by using appropriate keywords. The quality of the final studies was assessed using the NOS Checklist for case–control studies. The odds ratios (ORs) for each of the genetic models were pooled, and a subgroup analysis based on geographical region and types of infertility was carried out by the MetaGenyo online tool.

**Outcomes:**

Case–control studies including 18 and 2 studies about infertility in women and men, respectively, and 4 miscarriage studies were entered into the meta-analysis. The VDR gene TaqI polymorphism was associated with infertility susceptibility in women in the allele contrast [OR = 1.2065, 95% CI (1.0846–1.3421); *P* = 0.0005], Recessive model [OR = 1.3836, 95% CI (1.1197–1.7096); *P* = 0.002], Dominant model [OR = 1.2146, 95% CI (0.0484–1.4072); *P* = 0.009], Homozygote [OR = 1.4596, 95% CI (1.1627–1.8325); *P* = 0.001], and TT vs. Tt [OR = 1.2853, 95% CI (1.0249–1.6117); *P* = 0.029. ApaI and FokI gene polymorphisms were found to be significantly protective SNPs against women and men infertility in the Dominant model [OR = 0.8379, 95% CI (0.7039- 0.9975); *P* = 0.046] and Recessive model [OR = 0.421, 95% CI (0.1821–0.9767); *P* = 0.043], respectively. Sub-group meta-analysis showed a protection association of ApaI in dominant [OR = 0.7738, 95% CI = 0.6249–0.9580; *P* = 0.018] and AA vs. aa [OR = 0.7404, 95 CI% (0.5860–0.9353) *P* = 0.011725] models in PCOS subgroup, however, a negative association with idiopathic infertility was found in AA vs. Aa [OR = 1.7063, 95% CI (1.1039–2.6375); *P* = 0.016187] and Aa vs. aa [OR = 0.6069, 95% CI (0.3761–0.9792); *P* = 0.040754]. TaqI SNP was significantly associated with infertility in the African population and BsmI was associated with the disease mostly in the Asian population.

**Conclusion:**

This meta-analysis showed that the TaqI polymorphism may be linked to women’s infertility susceptibility. However, ApaI and FokI might be the protective SNPs against infertility in Women and men, respectively.

## Introduction

Infertility is a disease of the female or male reproductive system in which pregnancy does not occur after 12 months of regular unprotected sex [1]. This disease is a very common condition that affects between 48.5 and 186 million males and females worldwide, respectively. According to WHO, almost one out of six people of reproductive age experience infertility during their lifetime [2, 3]. Genetic, environmental, and some idiopathic factors are among the effective causes of infertility[4]. Male infertility is usually due to problems in the semen existence, the absence or low levels of sperm, or the abnormal shape and movement of sperm, and infertility in women is also caused by a range of abnormalities of the ovaries, uterus, fallopian tubes, the endocrine system, etc. [5]. Furthermore, Miscarriage is generally defined as the loss of a pregnancy before viability, which is considered the other complication of successful pregnancy [6]. It is estimated that 23 million miscarriages occur worldwide each year [7]. The short-term national economic cost of miscarriage in the UK was estimated at 471 million pounds annually in 2005 [8]. Physical consequences of miscarriage include bleeding or infection and psychological consequences such as increased risk of anxiety, depression, post-traumatic stress disorder, and suicide [9]. Its determinants include fetal genetic and chromosomal abnormalities, genital anatomy, endometrial pathology, hereditary thrombophilia, antiphospholipid syndrome, etc. Most of these factors are difficult to correct, but there are also controllable ones whose negative effects can be completely reduced before conception. These include nutritional deficiencies, including vitamin D (Vit D) deficiency [10, 11].

Vit D is a hormone that has a fundamental role in endocrine function, regulation of cell proliferation, and other metabolic pathways, such as pathways involved in the immune response [12]. Recent studies show the relationship between vitamin D deficiency and adverse pregnancy outcomes, including miscarriage [13–15]. Vit D is locally metabolized in the male reproductive system and the expression of Vitamin D receptor (VDR) has been shown in human testes and in ejaculated human sperms [16]. Studies have proven that Men who receive more diet and supplements produce sperm with less DNA damage [17]. Maternal Vit D deficiency is associated with many gynecological and obstetric diseases such as polycystic ovary syndrome, endometriosis, ovarian cancer, as well as gestational diabetes, which are all associated with reduced successful pregnancy. Preeclampsia and preterm labor are related, which can affect fertility [4, 18, 19]. Polycystic ovary syndrome (PCOS) is the most common endocrine metabolic disorder that affects 5 to 10% of women of reproductive age and is one of the common causes of ovulatory infertility [20]. The VDR gene is considered an important candidate gene for PCOS [21].

Since new research studies indicated the significance of vitamin D in the endocrine system and its relation not only with bone mineral density but also with certain cancers, autoimmune diseases, diabetes mellitus, depression, allergy, cardiovascular disease, pregnancy complications, infertility, and even frailty, vitamin D deficiency has just been identified as endemic to a variety of health consequences [22, 23]. The results of various studies taken together have shown that a variety of environmental and genetic factors influence vitamin D status variations. Studying the genetic basis of vitamin D metabolism, however, has brought to light the significance of multiple genes, including CG, DHCR1, CYP2R1, CYP24A1, and VDR [24]. By interacting with the vitamin D receptor (VDR), a member of the superfamily of steroid/thyroid hormone receptors, 1,25(OH)2D3, the active form of vitamin D, affects the transcriptional activation and repression of several target genes [25]. The VDR gene has many single-nucleotide polymorphisms (SNPs), which have been linked to a variety of physiological and pathological characteristics including different pregnancy complications in numerous populations [26–28]. VDR gene polymorphisms most likely have an impact on the expression and function of VDR [29].VDRs are found in the endometrium, placenta, decidual cells, ovarian granulosa cells, fallopian tube epithelium, pituitary gland, and hypothalamus. [30] Expression of the VDR in the placenta and decidua, which probably has an active role in the local autocrine and paracrine response, suggests that the local synthesis of Vit D potentially modulates placental function and fetal growth. Therefore, VDR gene function could be influenced by several factors such as genetic polymorphism that might related to susceptibility to fertility problems [31, 32]. The most intensively studied VDR polymorphisms are FokI (rs2228570), TaqI (rs731236), BsmI (rs1544410), and ApaI (rs7975232) variants. The association of these polymorphisms with different types of infertility complications including PCOS, endometriosis, miscarriage, etc. has been investigated in single studies, with conflicting results. Considering the above-mentioned observations, our meta-analysis study aimed to more powerfully and comprehensively assess the association between four VDR polymorphisms (rs2228570, rs1544410, rs7975232, rs731236) and infertility and miscarriage in different populations and geographical regions by conducting a systematic review.

## Methods

**Fig. 1:**
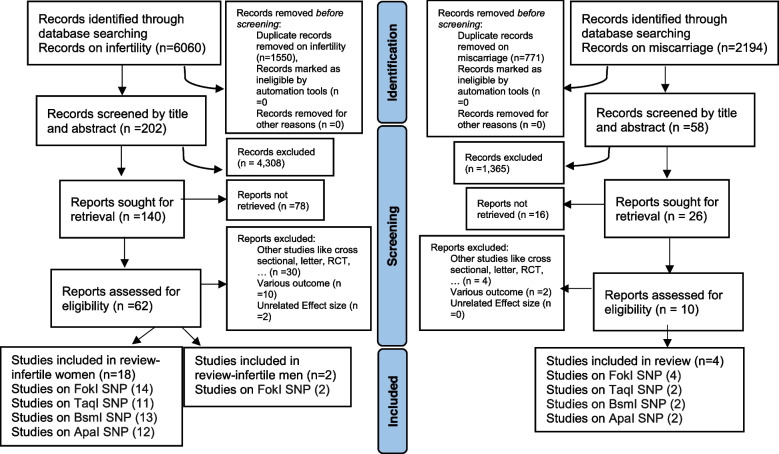
Flow diagram of study selection process

This systematic review and meta-analysis were conducted based on the preferred reporting items for systematic reviews and meta-analyses (PRISMA) guidelines (Fig. 1) [33]. The PROSPERO registration number and the published protocol were CRD42023416535.

**Fig. 2:**
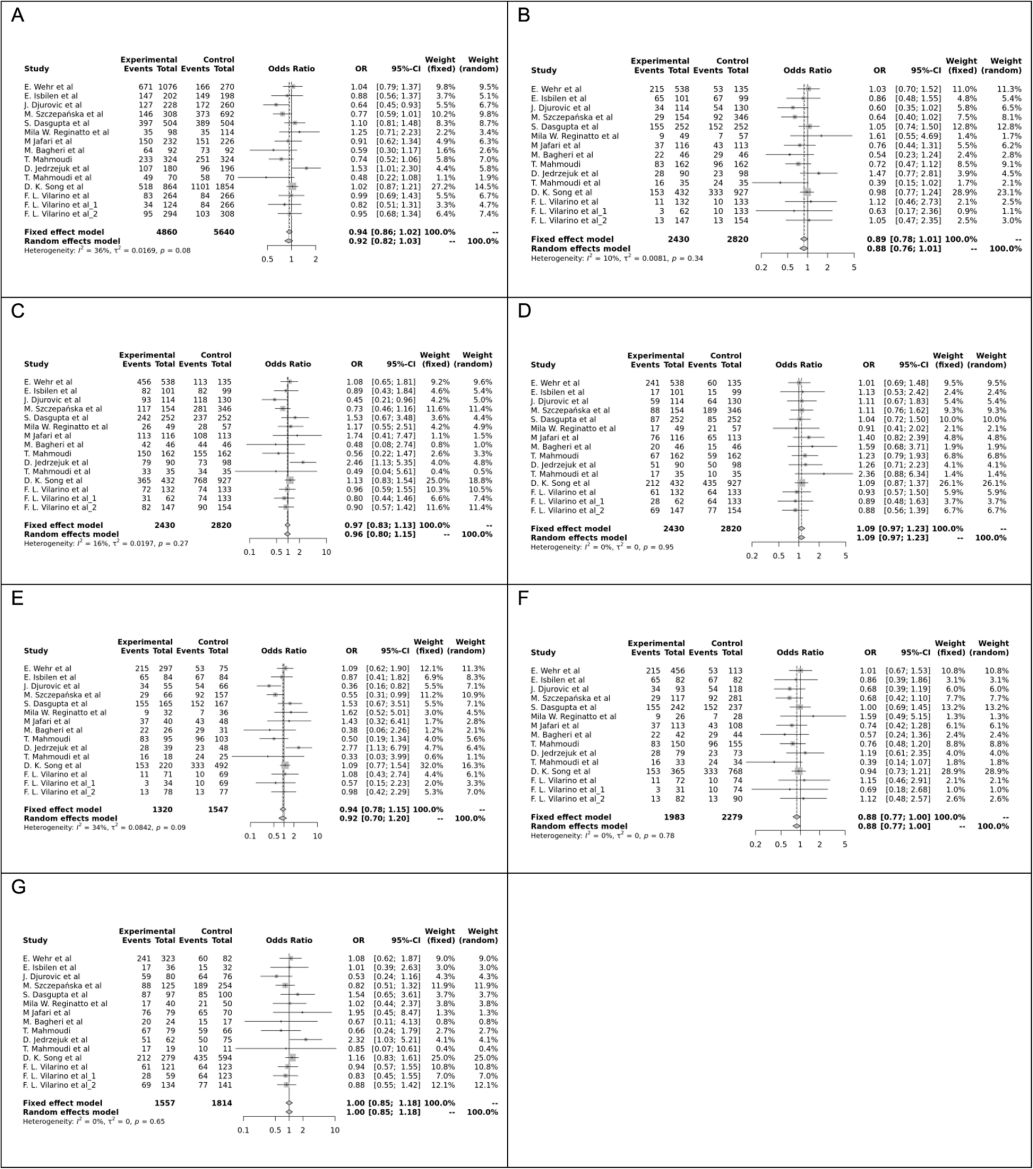
Forest plot of different genetic models in infertile women in FokI (rs2228570) SNP A; Allele contrast (F vs. f), B; Recessive model, C; Dominant model, D; Over dominant model, E; Homozygote model, F; FF vs. Ff model, G; Ff vs. ff model

**Fig. 3:**
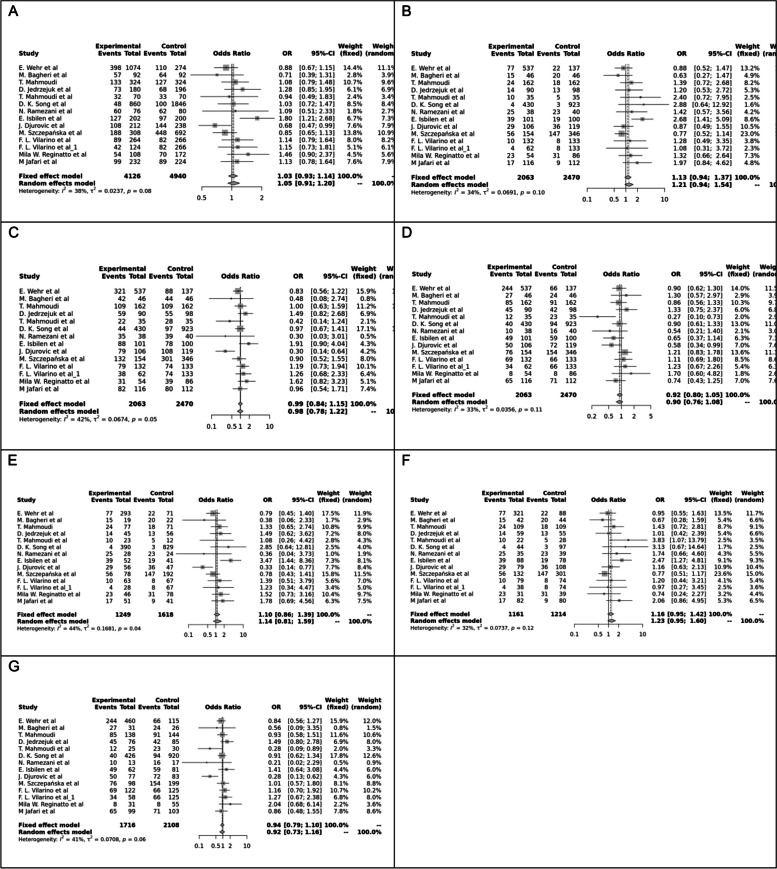
Forest plot of different genetic models in infertile women in BsmI (rs1544410) SNP A; Allele contrast (B vs. b), B; Recessive model, C; Dominant model, D; Over dominant model, E; Homozygote model, F; BB vs. Bb model, G; Bb vs. bb model

**Fig. 4:**
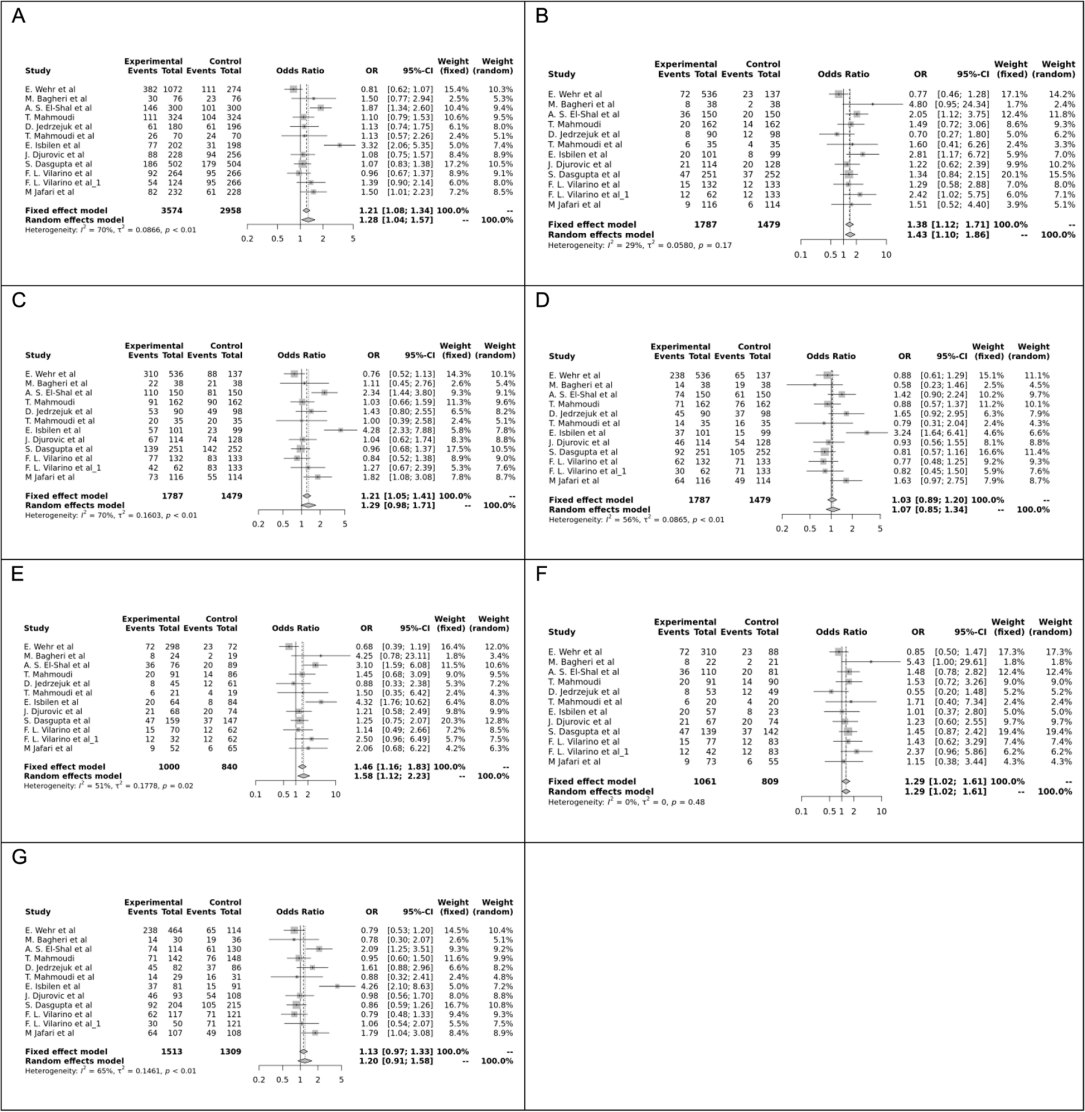
Forest plot of different genetic models in infertile women in TaqI (rs731236) SNP A; Allele contrast (T vs. t), B; Recessive model, C; Dominant model, D; Over dominant model, E; Homozygote model, F; TT vs. Tt model, G; Tt vs. tt model

**Fig. 5:**
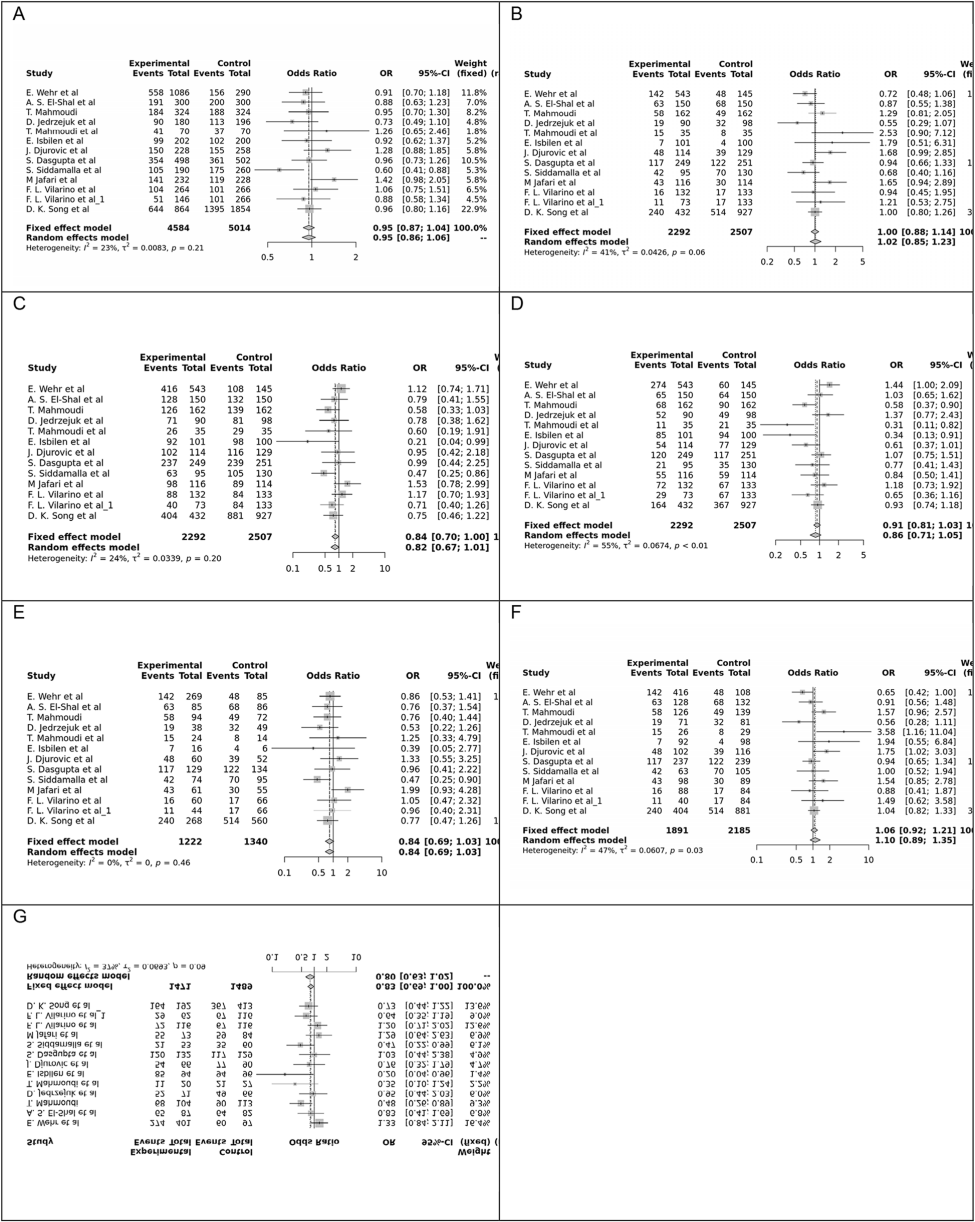
Forest plot of different genetic models in infertile women in ApaI (rs7975232) SNP A; Allele contrast (A vs. a), B; Recessive model, C; Dominant model, D; Over dominant model, E; Homozygote model, F; AA vs. Aa model, G; Aa vs. aa model

**Fig. 6:**
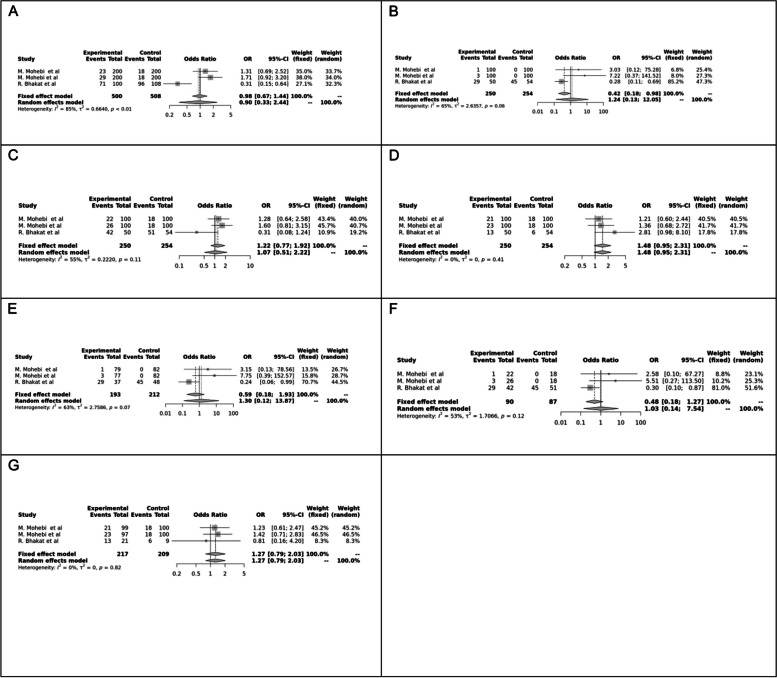
Forest plot of different genetic models in infertile men in FokI (rs2228570) SNP A; Allele contrast (F vs. f), B; Recessive model, C; Dominant model, D; Over dominant model, E; Homozygote model, F; FF vs. Ff model, G; Ff vs. ff model

**Fig. 7:**
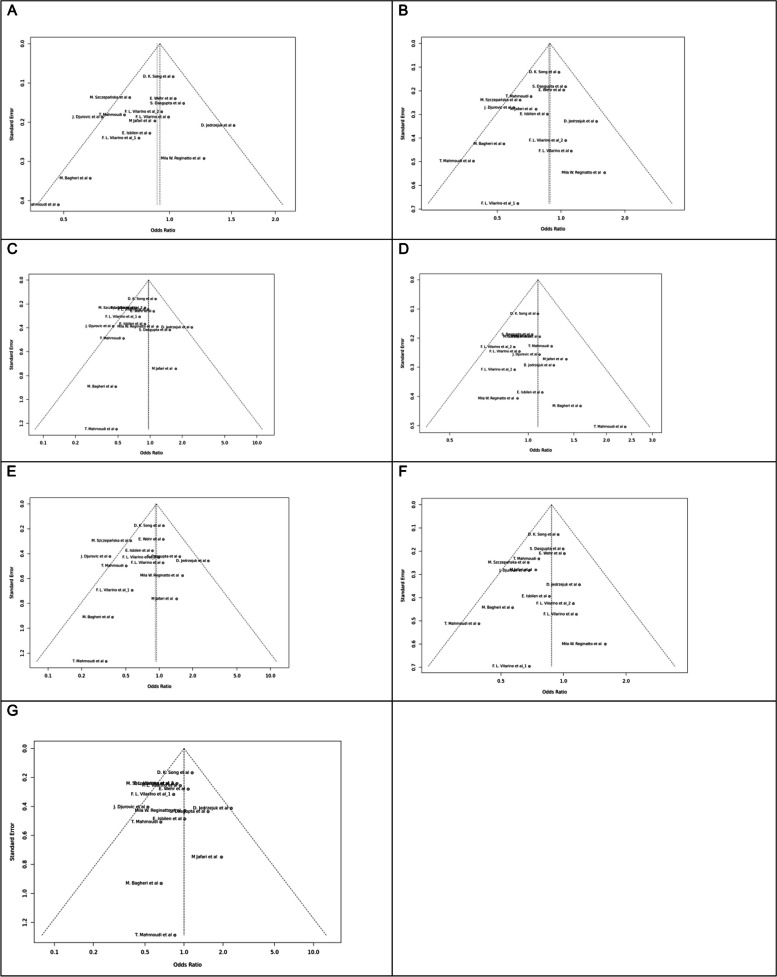
Funnel plot of different genetic models in infertile women in FokI (rs2228570) SNP A; Allele contrast (F vs. f), B; Recessive model, C; Dominant model, D; Over dominant model, E; Homozygote model, F; FF vs. Ff model, G; Ff vs. ff model

**Fig. 8:**
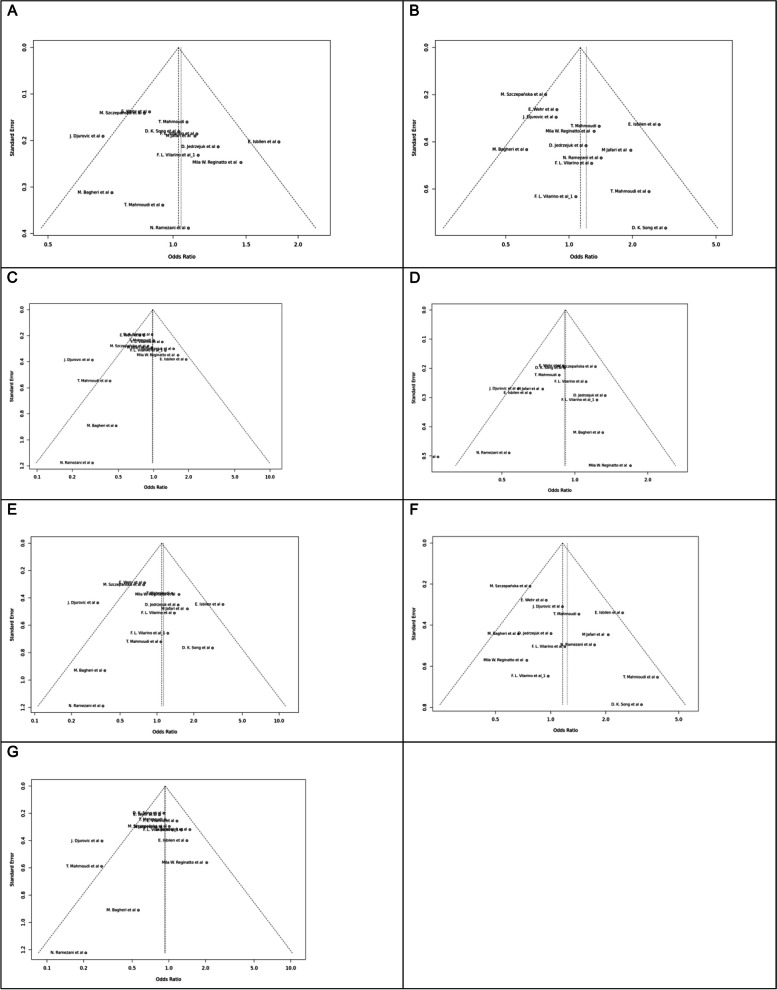
Funnel plot of different genetic models in infertile women in BsmI (rs1544410) SNP A; Allele contrast (B vs. b), B; Recessive model, C; Dominant model, D; Over dominant model, E; Homozygote model, F; BB vs. Bb model, G; Bb vs. bb model

**Fig. 9:**
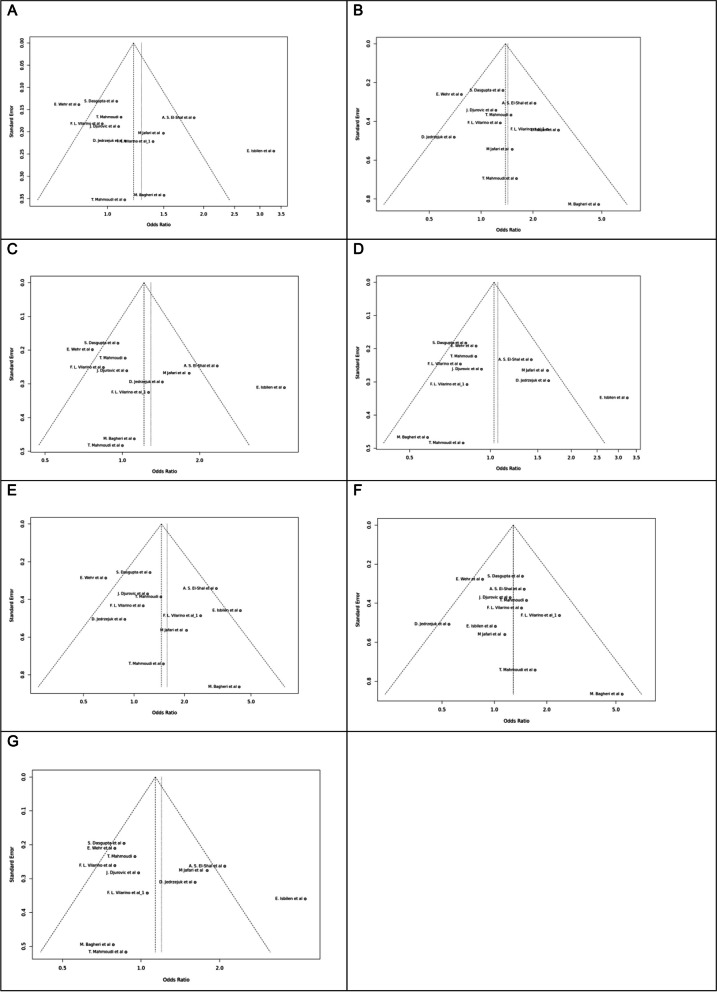
Funnel plot of different genetic models in infertile women in TaqI (rs731236) SNP A; Allele contrast (T vs. t), B; Recessive model, C; Dominant model, D; Over dominant model, E; Homozygote model, F; TT vs. Tt model, G; Tt vs. tt model

**Fig. 10:**
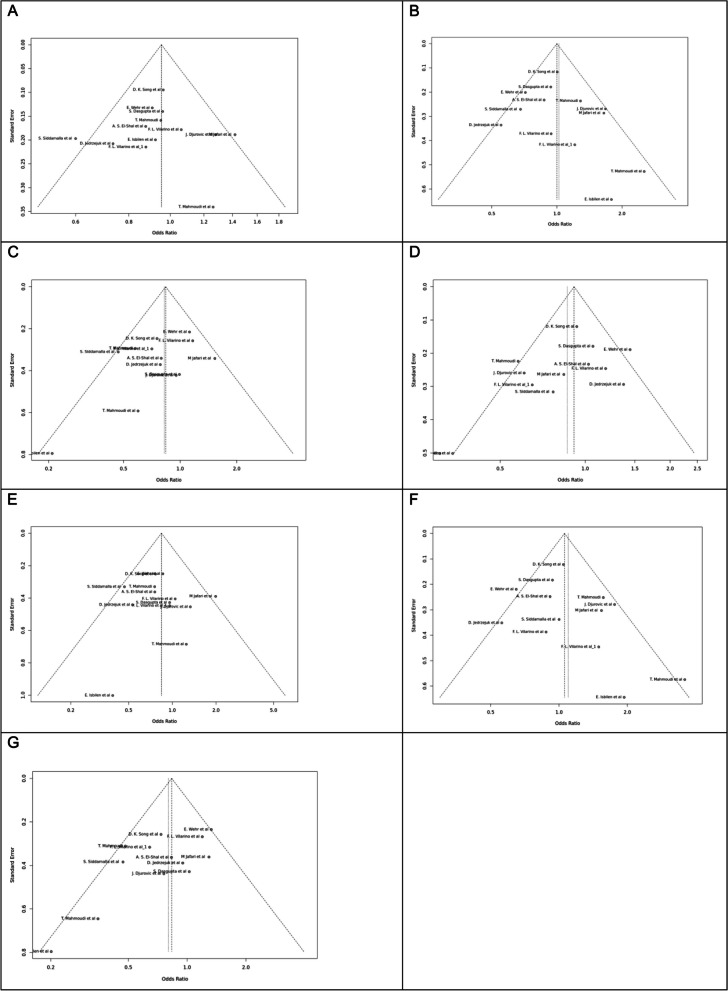
Funnel plot of different genetic models in infertile women in ApaI (rs7975232) SNP A; Allele contrast (A vs. a), B; Recessive model, C; Dominant model, D; Over dominant model, E; Homozygote model, F; AA vs. Aa model, G; Aa vs. aa model

**Fig. 11:**
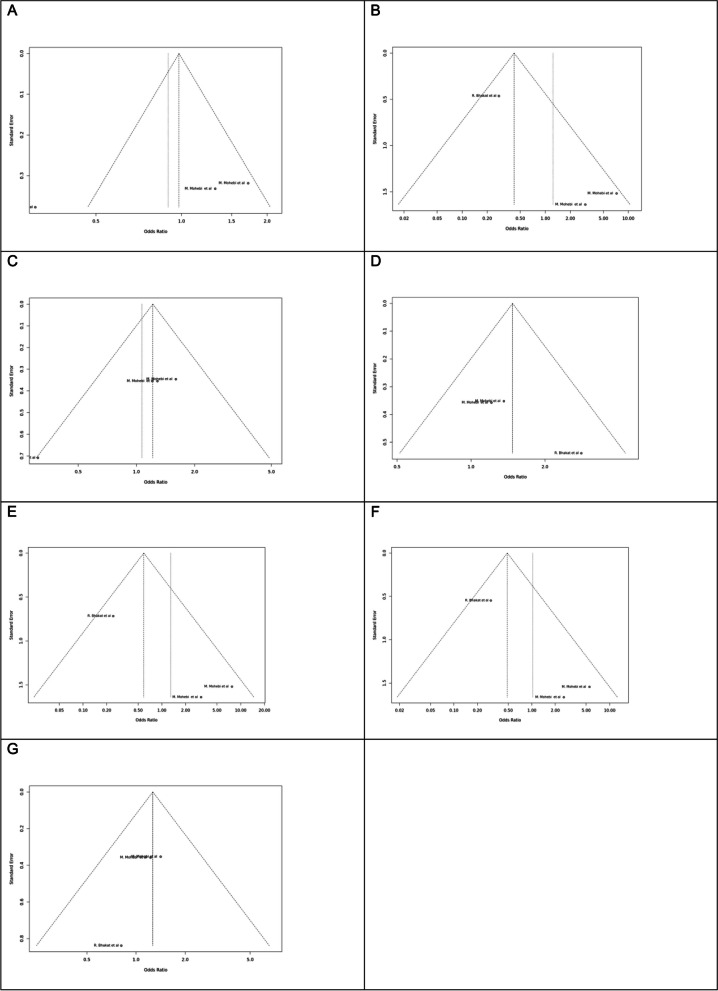
Funnel plot of different genetic models in infertile men in FokI (rs2228570) SNP A; Allele contrast (F vs. f), B; Recessive model, C; Dominant model, D; Over dominant model, E; Homozygote model, F; FF vs. Ff model, G; Ff vs. ff model

**Fig. 12:**
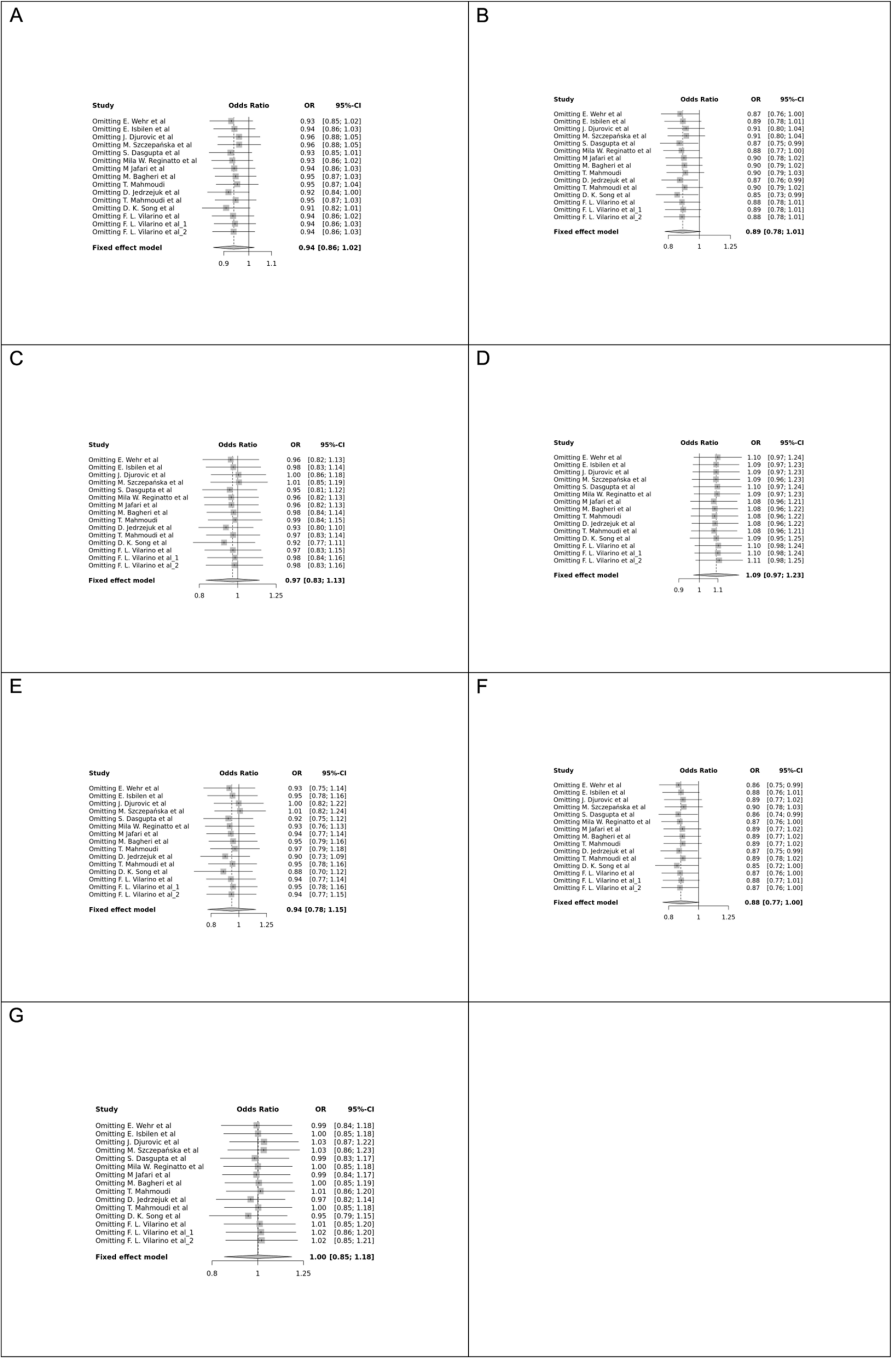
Sensitivity plot of different genetic models in infertile women in FokI (rs2228570) SNP A; Allele contrast (F vs. f), B; Recessive model, C; Dominant model, D; Over dominant model, E; Homozygote model, F; FF vs. Ff model, G; Ff vs. ff model

**Fig. 13:**
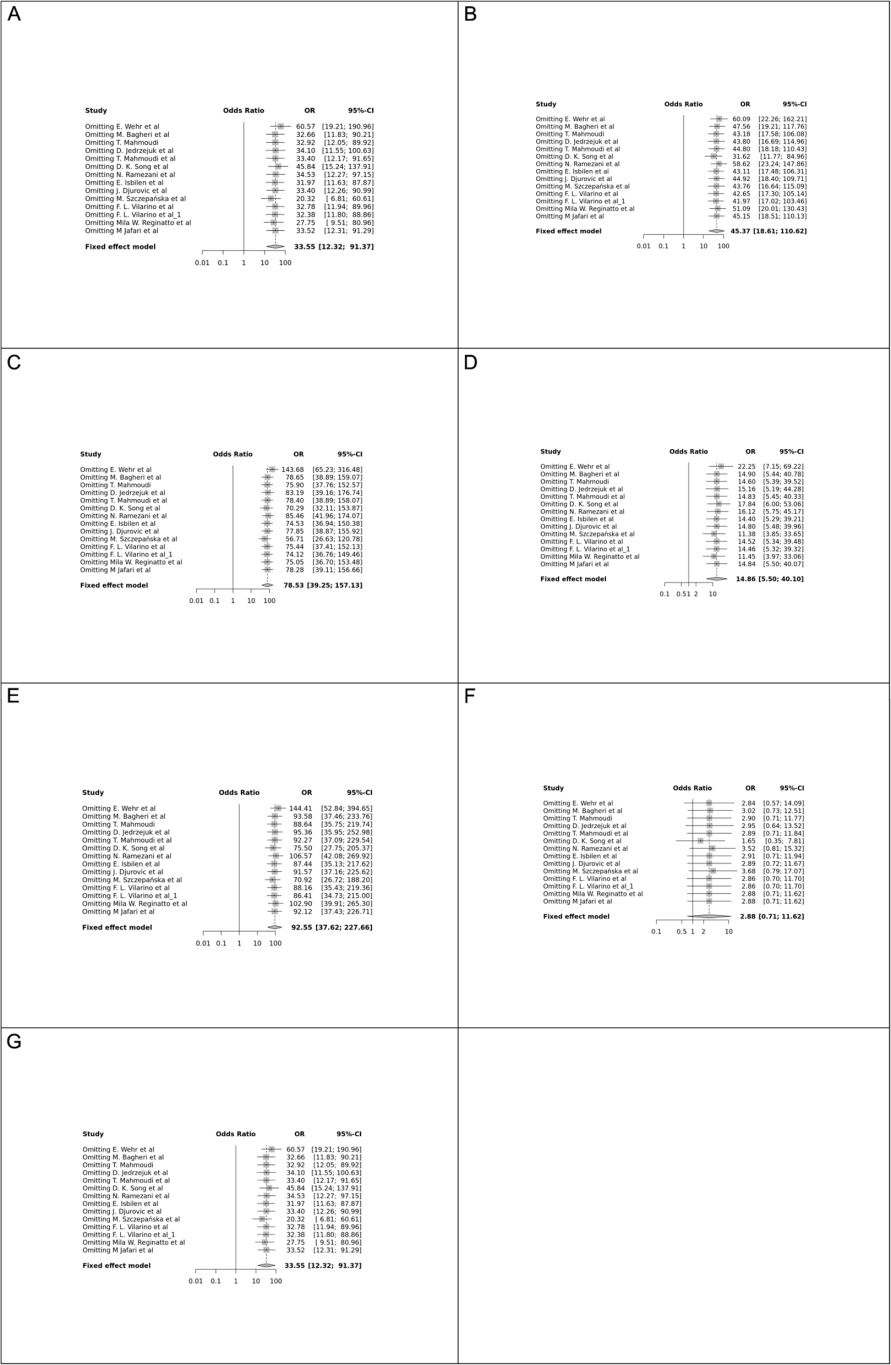
Sensitivity plot of different genetic models in infertile women in BsmI (rs1544410) SNP A; Allele contrast (B vs. b), B; Recessive model, C; Dominant model, D; Over dominant model, E; Homozygote model, F; BB vs. Bb model, G; Bb vs. bb model

**Fig. 14:**
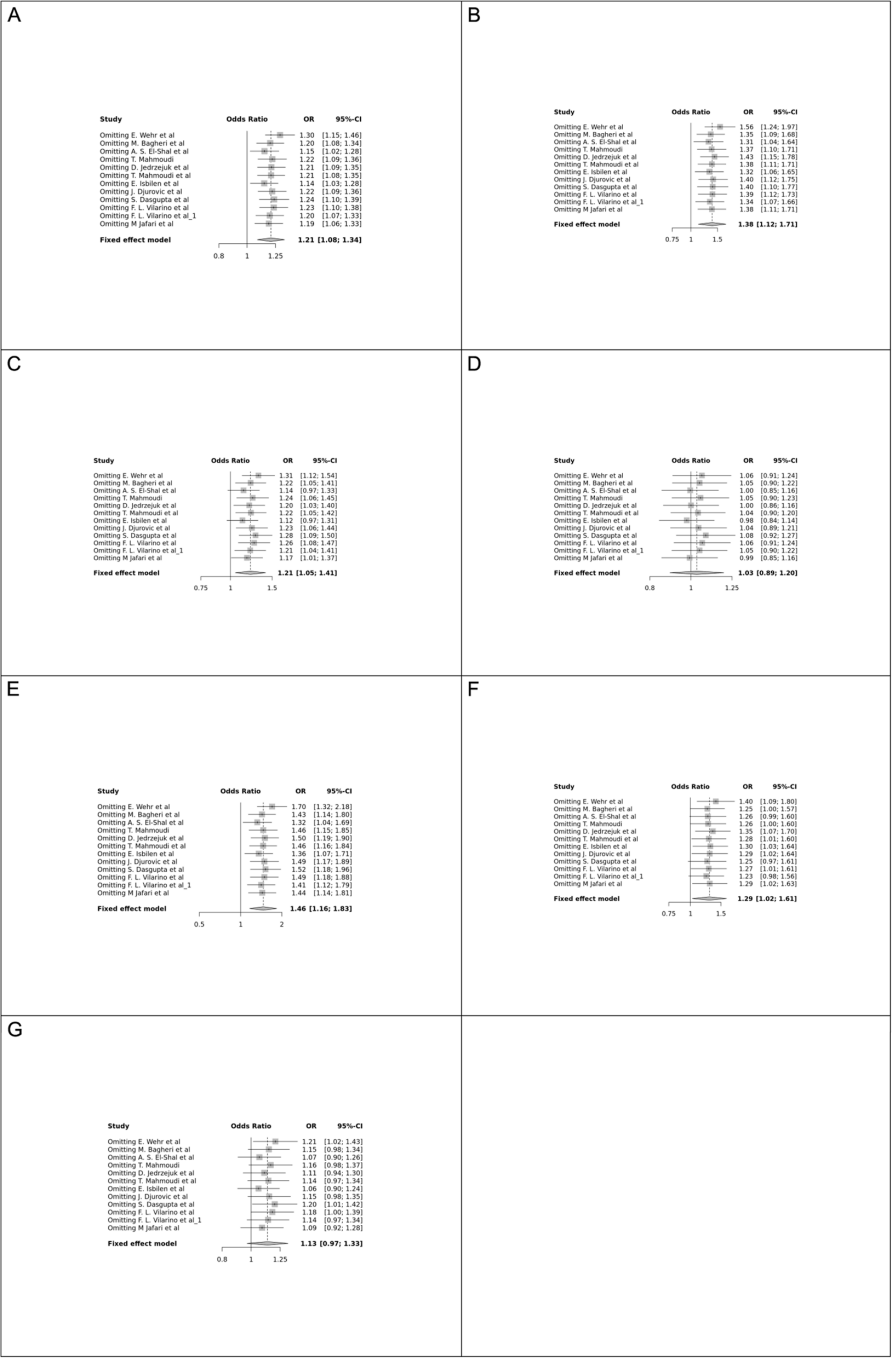
Sensitivity plot of different genetic models in infertile women in TaqI (rs731236) SNP A; Allele contrast (T vs. t), B; Recessive model, C; Dominant model, D; Over dominant model, E; Homozygote model, F; TT vs. Tt model, G; Tt vs. tt model

**Fig. 15:**
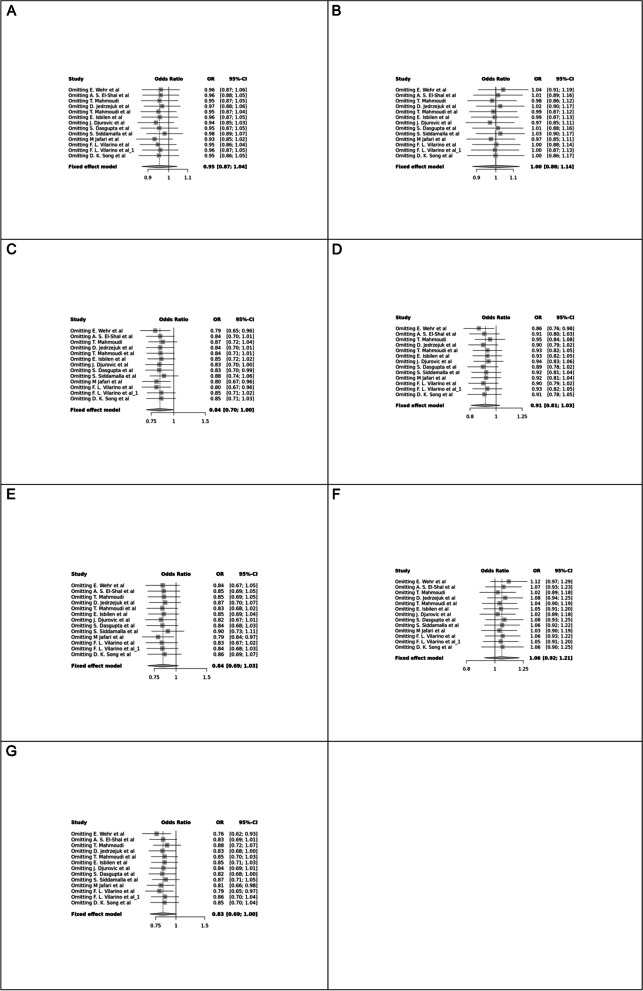
Sensitivity plot of different genetic models in infertile women in ApaI (rs7975232) SNP A; Allele contrast (A vs. a), B; Recessive model, C; Dominant model, D; Over dominant model, E; Homozygote model, F; AA vs. Aa model, G; Aa vs. aa model

**Fig. 16:**
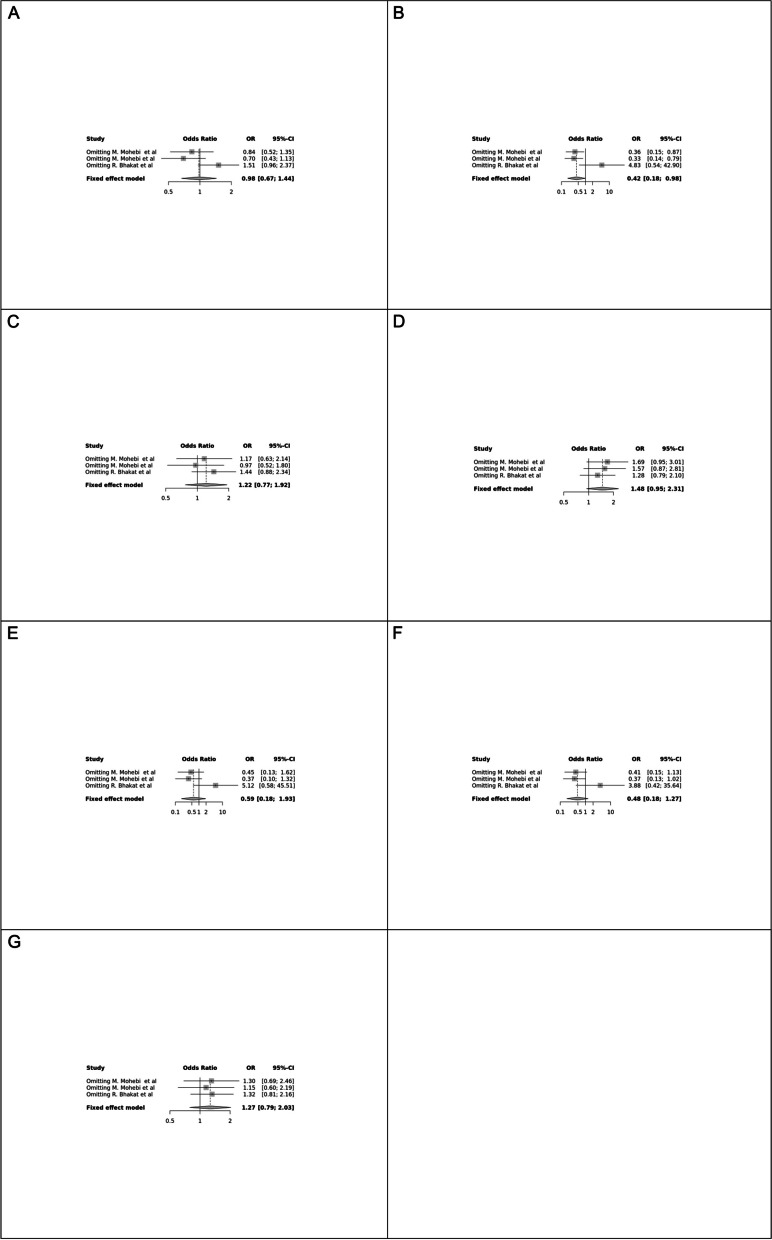
Sensitivity plot of different genetic models in infertile men in FokI (rs2228570) SNP A; Allele contrast (F vs. f), B; Recessive model, C; Dominant model, D; Over dominant model, E; Homozygote model, F; FF vs. Ff model, G; Ff vs. ff model

### Search strategy and screening process

For this meta-analysis, we accessed several international databases, including PubMed (Medline), Web of Science, and Scopus. These databases were searched for literature published up to January 2023, using specific search terms and their synonyms: "Infertility," "miscarriage," "VDR" or "vitamin D receptor," and "Polymorphism." Furthermore, we conducted a manual search within these databases, carefully examining the references of relevant studies, and also looked through grey literature to identify any additional related studies that might not have been captured through the initial database search. To ensure thoroughness and accuracy, the screening process was carried out independently by two authors (AM, MA). In cases where there were disagreements, they were resolved through discussion and consensus with a third author (YM). This rigorous approach helped maintain the integrity of the study selection process and ensured that all relevant studies were included for analysis.

### Eligibility criteria

Eligible studies were limited to those [1] case–control studies whose main purpose was to determine the association between VDR gene polymorphisms ApaI, BsmI, TaqI, and FokI and the risk of infertility and miscarriage, (2) studies that have reported the frequencies of genotypes or alleles by comparing at least two groups, a group including Infertility or miscarriage against healthy groups, (3) and, studies report odds ratio (ORs) and 95% confidence interval (CIs). Exclusion criteria included (1) all other types of studies including cohorts, cross-sectional, case reports, case series, letters to the editor, reports, clinical trial studies, and review studies, (2) Case–control studies without reporting inclusion criteria, (3) Repetitive and non-English language studies.

### Data extraction

The process of data extraction involved utilizing a structured form designed to gather essential information from each included study. This information encompassed various key aspects, such as the author's name, study location, publication date, ethnicity of the participants, mean age of the study population, sample size, genotyping methods employed, as well as the number of cases and controls pertaining to VDR gene polymorphisms.

### Risk of *Bias*

The Newcastle–Ottawa Quality Assessment Scale (NOS) checklist was used with the purpose of methodological quality and risk evaluation of non-randomized studies in Meta-Analysis. The NOS consists of eight items divided into three categories: Selection of cases and controls, comparability of them, and Ascertainment of exposure. Items can be awarded a maximum of one star for each one within the two first categories and a maximum of two stars for Comparability. The scoring ranged from zero to nine. A score of 6 and above indicates the high quality of the study[34].

### Statistical analysis

The control genotype distribution was assessed by the Hardy–Weinberg equilibrium (HWE) (*p* < 0.05 was considered meaningful). Calculation of ORs and 95% confidence intervals (CIs) in seven different genetic models was used to estimate its effect on a forest plot and the strength of the relationship between VDR polymorphisms and the risk of infertility and miscarriage. genetic models of polymorphisms are discussed in the following: TaqI —allele contrast (t vs. T), recessive model (tt vs. Tt + TT), dominant model (tt + Tt vs. TT), over dominant (Tt vs. TT + tt), tt vs. TT model, TT vs. Tt model, Tt vs. tt model; FokI — allele contrast (f vs. F), recessive model (ff vs. Ff + FF), dominant model (ff + Ff vs. FF), over-dominant model (Ff vs. FF + ff), ff vs. FF model, FF vs. Ff model, Ff vs. ff model; ApaI —allele contrast (a vs. A), recessive model (aa vs. Aa + AA), dominant model (aa + Aa vs. AA), over dominant model (Aa vs. AA + aa), aa vs. AA model, AA vs. Aa model, Aa vs. aa model; BsmI —allele contrast (b vs. B), recessive model (bb vs. Bb + BB), dominant model (bb + Bb vs. BB), over dominant model (Bb vs. BB + bb), bb vs. BB model, BB vs. Bb model, Bb vs. bb model. Also, heterogeneity between studies was assessed by Q Cochrane tests and I^2^. To test Publication biases, funnel plots and Egger’s test (*p* < 0.05) were used. All statistical analyses were done using MetaGenyo; a web tool to conduct a meta-analysis of genetic association studies [35]. The forest plot and funnel plot pertaining to all examined polymorphisms are depicted in (Figs. 2, 3, 4, 5, 6, 7, 8, 9, 10, 11, 12, 13, 14, 15 and 16).

## Results

### Description of Studies

Out of a total of 6060 and 2194 relevant citations on infertility and miscarriage, respectively, 5930 references remained after eliminating duplicates. Following title and abstract screening, and full-text review, 24 studies meeting the search criteria were identified, comprising 3424 cases and 3697 controls (refer to Tables 1, 2 and 3). Notably, the total cases and controls were categorized based on the methodology of the studies rather than individual SNP analysis. Among these studies, 18 investigated the association of VDR genetic polymorphisms with female infertility, 2 with male infertility, and 4 with miscarriage. Tables 4, 5, and 6 summarizes the characteristics and genotype frequencies of the included studies.

**Table 1:** Characteristics of studies included in the meta-analysis (infertile women)

Study author	Year	Country	Continent	Ethnicity	Disease	Case/control in each SNP	Age case/control (Mean)	Genotyping Method	NOS score
FokI (rs2228570)									
E. Wehr et al	2011	Austria	Europe		PCOS	538/135	23–31/ 26–36	NucleoSpin Blood method	8
E. Isbilen et al	2020	Turkey	Asia		Idiopathic infertility	101/99	27.45 ± 5.75/ 29.91 ± 4.98	PCR–RFLP	7
J. Djurovic et al	2018	Sebria	Europe		Idiopathic infertility	114/130		PCR–RFLP	6
M. Szczepańska et al	2015	Poland	Europe	Caucasian	Endometriosis-associated infertility	154/346	20–42/ 19–39	PCR–RFLP	8
S. Dasgupta et al	2015	India	Asia		PCOS	252/252		PCR–RFLP	9
Mila W. Reginatto et al	2018	Brazil	America		Idiopathic infertility	49/57	35 ± 0.5/ 44 ± 0.9	TaqMan qPCR and Sanger sequencing	8
M Jafari et al	2021	Iran	Asia		Endometriosis-associated infertility	116/113	32 ± 12/ 31 ± 14	ARMS-PCR and PCR–RFLP	9
M. Bagheri et al	2012	Iran	Asia		PCOS	46/46	26.58 ± 3.33/ 28.24 ± 5.25	PCR–RFLP	8
T. Mahmoudi et al	2009	Iran	Asia		PCOS	162/162	28.92 ± 0.41/ 29.91 ± 0.58	PCR	9
D. Jedrzejuk et al	2015	Poland	Europe	Caucasian	PCOS	90/98		PCR and mini-sequencing	9
T. Mahmoudi et al	2015	Iran	Asia		PCOS	35/35	19–42/ 19–44	PCR–RFLP	8
D. K. Song et al	2019	Korea	Asia		PCOS	432/927	24 ± 5/ 27 ± 5		8
F. L. Vilarino et al	2011	Brazil	America		Endometriosis-associated infertility	132/133	35.1 ± 3.9/ 39.7 ± 3.2	PCR–RFLP	7
F. L. Vilarino et al	2011	Brazil	America		Idiopathic infertility	62/133	35.7 ± 5.0/ 39.7 ± 3.2	PCR–RFLP	7
F. L. Vilarino et al	2011	Brazil	America		Endometriosis-associated infertility	147/154		PCR–RFLP	6
TaqI (rs731236)									
E. Wehr et al	2011	Austria	Europe		PCOS	536/137	23–31/ 26–36	NucleoSpin Blood method	8
M. Bagheri et al	2013	Iran	Asia		PCOS	38/38	26.03 ± 4.98/ 27.18 ± 4.95	PCR–RFLP	8
A. S. El-Shal et al	2013	Egypt	Africa	Caucasian	PCOS	150/150	29.8 ± 5.6/ 29.3 ± 6.2	PCR–RFLP	9
T. Mahmoudi et al	2009	Iran	Asia		PCOS	162/162	28.92 ± 0.41/ 29.91 ± 0.58	PCR	9
D. Jedrzejuk et al	2015	Poland	Europe	Caucasian	PCOS	90/98		PCR and mini-sequencing	9
T. Mahmoudi et al	2015	Iran	Asia	_	PCOS	35/35	19–42/19–44	PCR–RFLP	8
E. Isbilen et al	2020	Turkey	Asia		Idiopathic infertility	101/99	27.45 ± 5.75/ 29.91 ± 4.98	PCR–RFLP	7
J. Djurovic et al	2018	Sebria	Europe		Idiopathic infertility	114/128		PCR–RFLP	6
S. Dasgupta et al	2015	India	Asia		PCOS	251/252		PCR–RFLP	9
F. L. Vilarino et al	2011	Brazil	America		Endometriosis-associated infertility	132/133	35.1 ± 3.9/ 39.7 ± 3.2	PCR–RFLP	7
F. L. Vilarino et al	2011	Brazil	America		Idiopathic infertility	62/133	35.7 ± 5.0/ 39.7 ± 3.2	PCR–RFLP	7
M Jafari et al	2021	Iran	Asia		Endometriosis-associated infertility	116/114	32 ± 12/ 31 ± 14	ARMS-PCR and PCR–RFLP	9
BsmI (rs1544410)									
E. Wehr et al	2011	Austria	Europe		PCOS	537/137	23–31/ 26–36	NucleoSpin Blood method	8
M. Bagheri et al	2012	Iran	Asia		PCOS	46/46	26.58 ± 3.33/ 28.24 ± 5.25	PCR–RFLP	8
T. Mahmoudi et al	2009	Iran	Asia		PCOS	162/162	28.92 ± 0.41/ 29.91 ± 0.58	PCR	9
D. Jedrzejuk et al	2015	Poland	Europe	Caucasian	PCOS	90/98		PCR and mini-sequencing	9
T. Mahmoudi et al	2015	Iran	Asia		PCOS	35/35	19–42/ 19–44	PCR–RFLP	8
D. K. Song et al	2019	Korea	Asia		PCOS	430/923	24 ± 5/ 27 ± 5	-	8
N. Ramezani et al	2020	Iran	Asia		PCOS	38/40	28.58 ± 5.83/ 31.34 ± 5.5	PCR–RFLP	8
E. Isbilen et al	2020	Turkey	Asia		Idiopathic infertility	101/100	27.45 ± 5.75/ 29.91 ± 4.98	PCR–RFLP	7
J. Djurovic et al	2018	Serbia	Europe		Idiopathic infertility	106/119		PCR–RFLP	6
M. Szczepańska et al	2015	Poland	Europe	Caucasian	Endometriosis-associated infertility	154/346	20–42/ 19–39	PCR–RFLP	8
F. L. Vilarino et al	2011	Brazil	America		Endometriosis-associated infertility	132/133	35.1 ± 3.9/ 39.7 ± 3.2	PCR–RFLP	7
F. L. Vilarino et al	2011	Brazil	America		Idiopathic infertility	62/133	35.7 ± 5.0/ 39.7 ± 3.2	PCR–RFLP	7
Mila W. Reginatto et al	2018	Brazil	America		Idiopathic infertility	54/86	35 ± 0.5/ 44 ± 0.9	TaqMan qPCR and Sanger sequencing	8
M Jafari et al	2021	Iran	Asia		Endometriosis-associated infertility	116/112	32 ± 12/ 31 ± 14	ARMS-PCR and PCR–RFLP	9
ApaI (rs7975232)									
E. Wehr et al	2011	Austria	Europe		PCOS	543/145	23–31/ 26–36	NucleoSpin Blood method	8
A. S. El-Shal et al	2013	Egypt	Africa	Caucasian	PCOS	150/150	29.8 ± 5.6/ 29.3 ± 6.2	PCR–RFLP	9
T. Mahmoudi et al	2009	Iran	Asia		PCOS	162/162	28.92 ± 0.41/ 29.91 ± 0.58	PCR	9
D. Jedrzejuk et al	2015	Poland	Europe	Caucasian	PCOS	90/98		PCR and mini-sequencing-RFLP	9
T. Mahmoudi et al	2015	Iran	Asia		PCOS	35/35	19–42/ 19–44	PCR–RFLP	8
E. Isbilen et al	2020	Turkey	Asia		Idiopathic infertility	101/100	27.45 ± 5.75/ 29.91 ± 4.98	PCR–RFLP	7
J. Djurovic et al	2018	Serbia	Europe		Idiopathic infertility	114/129		PCR–RFLP	6
S. Dasgupta et al	2015	India	Asia		PCOS	249/251		PCR–RFLP	9
S. Siddamalla et al	2018	India	Asia		PCOS	95/130		PCR–RFLP	9
M Jafari et al	2021	Iran	Asia		Endometriosis-associated infertility	116/114	32 ± 12/ 31 ± 14	ARMS-PCR and PCR–RFLP	9
D. K. Song et al	2019	Korea	Asia		PCOS	432/927	24 ± 5/ 27 ± 5	-	8
F. L. Vilarino et al	2011	Brazil	America		Endometriosis-associated infertility	132/133	35.1 ± 3.9/ 39.7 ± 3.2	PCR–RFLP	7
F. L. Vilarino et al	2011	Brazil	America		Idiopathic infertility	72/133	35.7 ± 5.0/ 39.7 ± 3.2	PCR–RFLP	7

*NR* not reported, *PCR* Polymerase chain reaction, *RFLP* restriction fragment length polymorphism

**Table 2:** Characteristics of studies included in the meta-analysis (infertile men)

Study author	**Year**	**Country**	**Continent**	**Ethnicity**	**Disease**	**Total cases/controls**	**Case/control in each SNP**	**Age case/control** **(Mean)**	**Genotyping Method**	**NOS score**
FokI (rs2228570)										
M. Mohebi et al	2016	Iran	Asia		infertile men	100/100	100/100	39.1 ± 4.7/ 39.4 ± 5.07	PCR–RFLP	8
M. Mohebi et al	2016	Iran	Asia		infertile men	100/100	100/100	39.3 ± 4.8/ 39.4 ± 5.07	PCR–RFLP	8
R. Bhakat et al	2017	India	Asia		infertile men	50/54	50/54	28.78 ± 4.92/-	PCR–RFLP	7

**Table 3:** Characteristics of studies (miscarriage)

Study author	Year	Country	Continent	Ethnicity	Disease	Total cases/controls	Case/control in each SNP	Age case/control (Mean)	Genotyping Method	NOS score
**TaqI (rs731236)**										
A Barisic et al	2019	Slovenia and Croatia	Europe		Recurrent pregnancy loss (RPL) loss of two or more pregnancies before 24 weeks of gestation	160/160	160/160		PCR–RFLP	7
**ApaI (rs7975232)**										
D. Liu et al	2021	China	Asia		Recurrent pregnancy loss (RPL) loss of two or more pregnancies before 24 weeks of gestation	75/83	71/49	20–45/ 20–45	sequencing PCR	8
**FokI (rs2228570)**										
V. E. Radzinsky et al	2021	Russia	Asia		Recurrent pregnancy loss (RPL) loss of two or more pregnancies before 24 weeks of gestation	43/77	43/76	18–41/ 18–41	RT-PCR	9
A Barisic et al	2019	Slovenia and Croatia	Europe		Recurrent pregnancy loss (RPL) loss of two or more pregnancies before 24 weeks of gestation	160/160	160/160		PCR–RFLP	7

**Table 4:** Distribution of genotype and allele among cases and controls (infertile women)

Study author	Cases					Controls					P-HWE	P-HWE adjusted
	**FF**	**Ff**	**ff**	**F**	**f**	**FF**	**Ff**	**ff**	**F**	**f**		
**FokI (rs2228570)**												
**E. Wehr et al**	82	241	215	405	671	22	60	53	104	166	0.473	0.683
**E. Isbilen et al**	19	17	65	55	147	17	15	67	49	149	0.00	0.551
**J. Djurovic et al**	21	59	34	101	127	12	64	54	88	172	0.257	0.325
**M. Szczepańska et al**	37	88	29	162	146	65	189	92	319	373	0.065	0.683
**S. Dasgupta et al**	10	87	155	107	397	15	85	152	115	389	0.501	0.583
**Mila W. Reginatto et al**	23	17	9	63	35	29	21	7	79	35	0.311	0.01
**M Jafari et al**	3	76	37	82	150	5	**65**	43	75	151	0.001	0.972
**M. Bagheri et al**	4	20	22	28	64	2	15	29	19	73	0.972	0.727
**T. Mahmoudi et al**	12	67	83	91	233	7	59	96	73	251	0.581	0.965
**D. Jedrzejuk et al**	11	51	28	73	107	25	50	23	100	96	0.836	0.972
**T. Mahmoudi et al**	2	17	16	21	49	1	10	24	12	58	0.972	0.768
**D. K. Song et al**	67	212	153	346	518	159	435	333	753	1101	0.407	0.678
**F. L. Vilarino et al**	60	61	11	181	83	59	64	10	182	84	0.190	0.475
**F. L. Vilarino et al**	31	28	3	90	34	59	64	10	182	84	0.190	0.475
**F. L. Vilarino et al**	65	69	13	199	95	64	77	13	205	103	0.126	0.473
**Study author**	**Cases**					**Controls**					**P-HWE**	**P-HWE adjusted**
	**TT**	**Tt**	**tt**	**T**	**t**	**TT**	**Tt**	**tt**	**T**	**t**		
**TaqI (rs731236)**												
**E. Wehr et al**	226	238	72	690	382	49	65	23	163	111	0.854	0.931
**M. Bagheri et al**	16	14	8	46	30	17	19	2	53	23	0.255	0.398
**A. S. El-Shal et al**	40	74	36	154	146	69	61	20	199	101	0.27	0.398
**T. Mahmoudi et al**	71	71	20	213	111	72	76	14	220	104	0.330	0.398
**D. Jedrzejuk et al**	37	45	8	119	61	49	37	12	135	61	0.237	0.398
**T. Mahmoudi et al**	15	14	6	44	26	15	16	4	46	24	0.931	0.931
**E. Isbilen et al**	44	37	20	125	77	76	15	8	167	31	0.000	0.000
**J. Djurovic et al**	47	46	21	140	88	54	54	20	162	94	0.296	0.398
**S. Dasgupta et al**	112	92	47	316	186	110	105	37	325	179	0.151	0.398
**F. L. Vilarino et al**	55	62	15	172	92	50	71	12	171	95	0.060	0.243
**F. L. Vilarino et al**	20	30	12	70	54	50	71	12	171	95	0.060	0.243
**M Jafari et al**	43	64	9	150	82	59	49	6	167	61	0.301	0.398
**Study author**	**Cases**					**Controls**					**P-HWE**	**P-HWE adjusted**
	**BB**	**Bb**	**bb**	**B**	**b**	**BB**	**Bb**	**bb**	**B**	**b**		
**BsmI (rs1544410)**												
**E. Wehr et al**	77	244	216	398	676	22	66	49	110	164	0.977	0.977
**M. Bagheri et al**	15	27	4	57	35	20	24	2	64	28	0.115	0.179
**T. Mahmoudi et al**	24	85	53	133	191	18	91	53	127	197	0.023	0.080
**D. Jedrzejuk et al**	14	45	31	73	107	13	42	43	68	128	0.591	0.747
**T. Mahmoudi et al**	10	12	13	32	38	5	23	7	33	37	0.059	0.119
**D. K. Song et al**	4	40	386	48	812	3	94	826	100	1746	0.851	0.917
**N. Ramezani et al**	25	10	3	60	16	23	16	1	62	18	0.352	0.493
**E. Isbilen et al**	39	49	13	127	75	19	59	22	97	103	0.070	0.122
**J. Djurovic et al**	29	50	27	108	104	36	72	11	144	94	0.003	0.017
**M. Szczepańska et al**	56	76	22	188	120	147	154	45	448	244	0.640	0.747
**F. L. Vilarino et al**	10	69	53	89	175	8	66	59	82	184	0.059	0.111
**F. L. Vilarino et al**	4	34	24	42	82	8	66	59	82	184	0.059	0.111
**Mila W. Reginatto et al**	23	8	23	54	54	31	8	47	70	102	0.000	0.000
**M Jafari et al**	17	65	34	99	133	9	71	32	89	135	0.006	0.004
**Study author**	**Cases**					**Controls**					**P-HWE**	**P-HWE adjusted**
	**AA**	**Aa**	**aa**	**A**	**a**	**AA**	**Aa**	**aa**	**A**	**a**		
**ApaI (rs7975232)**												
**E. Wehr et al**	127	274	142	528	558	37	60	48	134	156	0.043	0.113
**A. S. El-Shal et al**	22	65	63	109	191	18	64	68	100	200	0.624	0.737
**T. Mahmoudi et al**	36	68	58	140	184	23	90	49	136	188	0.073	0.137
**D. Jedrzejuk et al**	19	52	19	90	90	17	49	32	83	113	0.812	0.812
**T. Mahmoudi et al**	9	11	15	29	41	6	21	8	33	37	0.227	0.369
**E. Isbilen et al**	9	85	7	103	99	2	94	4	98	102	0.000	0.000
**J. Djurovic et al**	12	54	48	78	150	13	77	39	103	155	0.005	0.023
**S. Dasgupta et al**	12	120	117	144	354	12	117	122	141	361	0.014	0.048
**S. Siddamalla et al**	32	21	42	85	105	25	35	70	85	175	0.000	0.000
**M Jafari et al**	18	55	43	91	141	25	59	30	109	119	0.692	0.749
**D. K. Song et al**	28	164	240	220	644	46	367	514	459	1395	0.056	0.122
**F. L. Vilarino et al**	44	72	16	160	104	49	67	17	165	101	0.423	0.550
**F. L. Vilarino et al**	22	29	11	73	51	49	67	17	165	101	0.423	0.550

*P-HWE P* value for Hardy–Weinberg equilibrium

**Table 5:** Distribution of genotype and allele among cases and controls (infertile men)

Study author	cases					controls					P-HWE	P-HWE adjusted
	**FF**	**Ff**	**ff**	**F**	**f**	**FF**	**Ff**	**ff**	**F**	**f**		
**FokI (rs2228570)**												
**M. Mohebi et al**	1	21	78	23	177	0	18	82	18	182	0.322	0.322
**M. Mohebi et al**	3	23	74	29	171	0	18	82	18	182	0.322	0.322
**R. Bhakat et al**	8	13	29	29	71	3	6	45	12	96	0.001	0.003

**Table 6:** Distribution of genotype and allele among cases and controls (miscarriage)

**Study author**	C**ases**					C**ontrols**					**P-HWE**	**P-HWE adjusted**
	**FF**	**Ff**	**ff**	**F**	**f**	**FF**	**Ff**	**ff**	**F**	**f**		
**FokI (rs2228570)**												
**V. E. Radzinsky et al**	16	21	6	53	33	21	37	18	79	73	0.828	0.832
**A Barisic et al**	17	75	68	109	211	26	87	47	139	181	0.177	0.355
**Study author**	**Cases**					**Controls**					**P-HWE**	**P-HWE adjusted**
	**AA**	**Aa**	**aa**	**A**	**a**	**AA**	**Aa**	**aa**	**A**	**a**		
**ApaI (rs7975232)**												
**D. Liu et al**	48	20	3	116	26	30	17	2	77	21	0.832	0.832
**Study author**	**Cases**					**Controls**					**P-HWE**	**P-HWE adjusted**
	**TT**	**Tt**	**tt**	**T**	**t**	**TT**	**Tt**	**tt**	**T**	**t**		
**TaqI (rs731236)**												
**A Barisic et al**	23	64	73	110	210	34	67	59	135	185	0.073	0.293

### Association between VDR Genetic Polymorphisms and Infertility in Women

#### FokI (rs2228570) SNP

Fourteen studies involving 5,210 participants reported on the association between FokI SNP and female infertility. While a protective association was observed in the FF vs. Ff model (OR = 0.87, 95% CI = 0.76–1.00, *P* = 0.05), no significant association was found in other genetic models.

#### BsmI (rs1544410) SNP

Thirteen studies examined the association between BsmI SNP and infertility, with no significant correlation found in any of the genetic models assessed.

#### TaqI (rs731236) SNP

Eleven studies focused on TaqI SNP, reporting a positive association in some genetic models, including Allele contrast and Recessive model, but not in others.

#### ApaI (rs7975232) SNP

Twelve case–control studies evaluated the ApaI SNP, showing a protective association in the Dominant model and Aa vs. aa model, but no significant association in other genetic models.

### Association between VDR Genetic Polymorphisms and Infertility in Men

#### FokI (rs2228570) SNP

Two studies involving 404 participants investigated the association between FokI SNP and male infertility, revealing a protective association in the Recessive model.

### Association between VDR Genetic Polymorphisms and Miscarriage

Four studies examined the association between VDR genetic polymorphisms and miscarriage, evaluating TaqI, ApaI, FokI, and BsmI SNPs. Characteristics of these studies are summarized in Tables 7,8 and 9.

**Table 7:** Main results of pooled ORs in the meta-analysis of *VDR* gene polymorphisms (infertile women)

**Comparisons**		**Number of studies**	**Test of association**				**Test of heterogeneity**		**Publication bias**
			**OR**	**95% CI**	***p*-value**	**Model**	***p*-value**	**I^2**	***p*-value (Egger's test)**
**FokI (rs2228570)**									
**f vs. F**	Allele contrast	14	0.939	[0.861; 1.023]	0.151	Fixed	0.083	0.356	0.220
**ff vs. Ff + FF**	Recessive model	14	0.887	[0.780; 1.008]	0.067	Fixed	0.336	0.104	0.354
**ff + Ff vs. FF**	Dominant model	14	0.971	[0.831; 1.134]	0.712	Fixed	0.270	0.163	0.650
**Ff vs. FF + ff**	Over dominant	14	1.090	[0.970; 1.225]	0.145	Fixed	0.948	0.000	0.213
**ff vs. FF**	Homozygote	14	0.944	[0.777; 1.147]	0.564	Fixed	0.094	0.341	0.516
**FF vs. Ff**		14	0.876	[0.765; 1.003]	0.056	Fixed	0.776	0.000	0.439
**Ff vs. ff**		14	1.001	[0.849; 1.180]	0.985	Fixed	0.650	0.000	0.961
**TaqI (rs731236)**									
**t vs. T**	Allele contrast	11	1.206	[1.084; 1.342]	0.000	Fixed	0.000	0.704	0.148
**tt vs. Tt + TT**	Recessive model	11	1.383	[1.119; 1.709]	0.002	Fixed	0.165	0.285	0.160
**tt + Tt vs. TT**	Dominant model	11	1.214	[0.048; 1.407]	0.009	Fixed	0.000	0.698	0.270
**Tt vs. TT + tt**	Over dominant	11	1.0325	[0.892; 1.195]	0.668	Fixed	0.009	0.558	0.489
**tt vs. TT**	Homozygote	11	1.459	[1.162; 1.832]	0.001	Fixed	0.019	0.514	0.169
**TT vs. Tt**		11	1.285	[1.024; 1.611]	0.029	Fixed	0.479	0.000	0.350
**Tt vs. tt**		11	1.134	[0.969; 1.327]	0.115	Fixed	0.001	0.649	0.325
**BsmI (rs1544410)**									
**b vs. B**	Allele contrast	13	1.030	[0.929; 1.141]	0.570	Fixed	0.076	0.375	0.448
**bb vs. Bb + BB**	Recessive model	13	1.131	[0.938; 1.365]	0.195	Fixed	0.103	0.340	0.044
**bb + Bb vs. BB**	Dominant model	13	0.986	[0.844; 1.151]	0.859	Fixed	0.046	0.424	0.367
**Bb vs. BB + bb**	Over dominant	13	0.915	[0.795; 1.053]	0.216	Fixed	0.114	0.325	0.445
**bb vs. BB**	Homozygote	13	1.095	[0.864; 1.388]	0.449	Fixed	0.038	0.442	0.805
**BB vs. Bb**		13	1.161	[0.950; 1.419]	0.142	Fixed	0.116	0.323	0.085
**Bb vs. bb**		13	0.936	[0.794; 1.103]	0.434	Fixed	0.055	0.408	0.342
**ApaI (rs7975232)**									
**a vs. A**	Allele contrast	12	0.953	[0.872; 1.042]	0.299	Fixed	0.212	0.229	0.898
**aa vs. Aa + AA**	Recessive model	12	1.000	[0.879; 1.138]	0.992	Fixed	0.060	0.411	0.353
**aa + Aa vs. AA**	Dominant model	12	0.837	[0.703; 0.997]	0.046	Fixed	0.197	0.243	0.168
**Aa vs. AA + aa**	Over dominant	12	0.913	[0.807; 1.033]	0.149	Fixed	0.008	0.553	0.079
**aa vs. AA**	Homozygote	12	0.842	[0.688; 1.031]	0.097	Fixed	0.458	0.000	0.704
**AA vs. Aa**		12	1.056	[0.921; 1.210]	0.432	Fixed	0.031	0.468	0.260
**Aa vs. aa**		12	0.833	[0.691; 1.004]	0.055	Fixed	0.090	0.365	0.042

**Table 8:** Main results of pooled ORs in the meta-analysis of *VDR* gene polymorphisms (infertile men)

**Comparisons**		**Number of studies**	**Test of association**				**Test of heterogeneity**		**Publication bias**
			**OR**	**95% CI**	***p*-value**	**Model**	***p*-value**	**I^2**	***p*-value (Egger's test)**
**FokI (rs2228570)**									
**f vs. F**	Allele contrast	2	0.980	[0.667; 1.439]	0.918	Fixed	0.001	0.850	0.053
**ff vs. Ff + FF**	Recessive model	2	0.421	[0.182; 0.976]	0.043	Fixed	0.055	0.653	0.140
**ff + Ff vs. FF**	Dominant model	2	1.216	[0.768; 1.923]	0.402	Fixed	0.110	0.545	0.111
**Ff vs. FF + ff**	Over dominant	2	1.476	[0.945; 2.307]	0.086	Fixed	0.410	0.000	0.133
**ff vs. FF**	Homozygote	2	0.590	[0.181; 1.928]	0.383	Fixed	0.065	0.633	0.162
**FF vs. Ff**		2	0.484	[0.184; 1.273]	0.141	Fixed	0.117	0.533	0.143
**Ff vs. ff**		2	1.267	[0.790; 2.031]	0.325	Fixed	0.823	0.000	0.291

**Table 9:** Main results of pooled ORs in the meta-analysis of *VDR* gene polymorphisms (miscarriage)

**Comparisons**		**Number of studies**	**Test of association**				**Test of heterogeneity**		**Publication bias**
			**OR**	**95% CI**	***p*-value**	**Model**	***p*-value**	**I^2**	***p*-value (Egger's test)**
**FokI (rs2228570)**									
f vs. F	Allele contrast	4	1.238	[1.016; 1.508]	0.034	Fixed	0.187	0.373	0.934
ff vs. Ff + FF	Recessive model	4	1.476	[1.096; 1.986]	0.010	Fixed	0.626	0.000	0.657
ff + Ff vs. FF	Dominant model	4	1.139	[0.792; 1.638]	0.481	Fixed	0.164	0.411	0.418
Ff vs. FF + ff	Over dominant	4	0.774	[0.587; 1.020]	0.068	Fixed	0.886	0.000	0.144
ff vs. FF	Homozygote	4	1.462	[0.966; 2.212]	0.072	Fixed	0.188	0.372	0.693
FF vs. Ff		4	1.469	[1.074; 2.011]	0.016	Fixed	0.898	0.000	0.328
Ff vs. ff		4	0.992	[0.675; 1.456]	0.968	Fixed	0.294	0.190	0.379

### Heterogeneity, Publication *Bias*, and Sensitivity Analysis

Heterogeneity was observed in certain genetic models, particularly in the TaqI SNP. Egger's tests revealed no publication bias. The results of sensitivity analysis are presented in relevant charts.

## Discussion

This meta-analysis suggests that vitamin D receptor gene variations may play a role in infertility risk and outcome. The TaqI polymorphism may increase the susceptibility to infertility in women, possibly by affecting the implantation and placentation processes. The ApaI and FokI polymorphisms may have protective effects against infertility in women and men, respectively, possibly by modulating the immune system and the hormonal balance. These findings may have implications for the diagnosis, prevention, and treatment of infertility, as well as for the understanding of the molecular mechanisms of reproductive health. In the development of pregnancy problems, genetic variables have grown increasingly relevant. Previous research has linked VDR gene variations to infertility in women and men due to PCOS, endometriosis, preeclampsia, idiopathic infertility, and other causes [36, 37]. Serum 25-hydroxyvitamin D [25 (OH) D] has been found to inhibit VDR-mediated pathogenesis by modulating target gene expression [38]. The VDR gene is a potential gene for infertility because it controls several genes that participate in diverse molecular and cellular processes [39]. Recurrent miscarriages (RM), which occur at a rate of 1 to 3% of female reproductive age, are another major medical, social, and psychological complication associated with pregnancy. Although numerous pathways for the development of RM have already been discovered, the underlying causes of around 50% of patients remain unexplained [13, 14]. Nevertheless, the multifaceted etiology of this problem, including immune system irregularities and vitamin D inadequacy, has been recognized for some time. As a result, it seems that altered metabolism of the VD/VDR complex via immune response modulation might be significant in the pathogenesis of both spontaneous abortion and RM [40]. Furthermore, VDR is a receptor with a pleiotropic action on human cells. remarkably, the presence of polymorphic variations in the VDR gene may affect VDR activity [41]. Regarding the evaluation of the connection between VDR gene polymorphisms and infertility/recurrent miscarriage in numerous single studies in different populations, the findings are contradictory. Given the volume of data accumulated and the ambiguous role of VDR in the etiology of infertility/recurrent miscarriage in general, we decided to conduct a comprehensive meta-analysis of any published research on the association between the most studied VDR polymorphisms and any infertility/recurrent miscarriage.

The present study included a total of 22 articles and showed that the VDR gene TaqI polymorphism was associated with infertility susceptibility in women. ApaI and FokI gene polymorphisms were found to be significantly protective SNPs against women's and men's infertility. The published studies related to the association of selected VDR SNPs and recurrent miscarriage were not enough for meta-analysis, therefore, a systematic review was alone performed. The findings were consistent with prior research and may give an entirely novel biomarker in infertility/recurrent miscarriage with diverse etiologies [20, 42–45]. A subgroup analysis was also undertaken to investigate the possible significance of patient ethnicity or infertility etiology on the association between VDR polymorphisms and the risk of infertility. TaqI SNP was shown to be significantly connected with infertility in Africans, while BsmI was found to be associated with the disease mostly in Asians. This finding could be explained by genetic differences between ethnic groupings. Furthermore, due to the procedure of natural selection, functional variations in various groups may differ [26]. Furthermore, VDR ApaI (rs7975232) was found to be associated with infertility susceptibility in the PCOS subgroup, however, a protection association with idiopathic infertility was found.

VDR gene polymorphism could contribute to the pathophysiology of infertility by influencing gene expression and mRNA stability, and hence the cellular and molecular processes associated with infertility etiology. Nevertheless, these polymorphisms are mostly nonfunctional, linkage disequilibrium with another undiscovered functional variant of the VDR gene appears to be the most likely explanation for the observed association. We meta-analyzed the VDR gene TaqI, BsmI, FokI, ApaI polymorphisms, and women/men infertility for the first time.

The FokI SNP is the only VDR polymorphism leading to a VDR protein with a different structure. Furthermore, it is the only SNP that is not linked to any other VDR polymorphism, implying that it plays a distinct function [46]. The polymorphism, which is a C to T alteration, is located at the 5' end of the gene. This alteration results in a protein of a different size, a 424 amino acid (aa) variant encoded by the major allele form (ACG) and a 427 aa variant expressed by the minor allele form (ATG). The variations are thought to be functionally relevant, with the 424 aa VDR variant having higher transcriptional activity and being associated with lower circulating 25(OH) D levels than the 427 aa variant [46, 47]. Moreover, Yan et al. showed that women with RPL have lower levels of VDR expression in chorionic villi, decidua, and serum compared with normal pregnant women [48]. It has previously been suggested that CC genotype / 424 aa VDR variant has a higher frequency in women with RPL, which leads to lower circulating 25(OH) D levels, respectively. Several studies have demonstrated that high Vitamin D levels might protect against a variety of illnesses, including infertility and recurrent miscarriage. The idea has been suggested in several research that greater pre-diagnosis plasma levels of 25-hydroxy vitamin D, the predominant circulating form of vitamin D, is related to a significant decrease in pregnancy problems such as PCOS, endometriosis, infertility, and recurrent pregnancy loss [49–52]. Furthermore, comprehensive reviews and meta-analyses revealed a substantial reduction in total pregnancy outcomes in Vitamin D-deficient patients [53, 54]. Our finding revealed a marginally significant association of FokI SNP with infertility under the FF vs. Ff genetic model (OR = 0.8763, 95% CI [0.7651–1.0036], *P* = 0.05). This indicates that the FokI f allele might be a risk factor for infertility, future studies with larger sample sizes and considering other confounder variables still need to confirm these findings though.

The functional evaluation of the three significant non-coding VDR SNPs (Bsml, TaqI, and ApaI) examined in this meta-analysis revealed contradictory findings from prior studies regarding their biological implications. Even if these SNPs are nonfunctional, the impacts identified in this meta-analysis and other studies could be driven by other, actually important SNPs in significant LD located elsewhere in the VDR gene. Some studies aimed at characterizing differences in VDR expression for SNPs in the 3' end of the VDR gene found that the Bsml-ApaI-TaqI haplotype BAt (rs1544410-A/rs7975232-A/rs731236-C) had higher levels of VDR mRNA expression than the baT (rs1544410-G/rs7975232-C/rs731236-T). These SNPs could be implicated in gene expression control, specifically by mRNA stability modulation. To be more specific, the existence of the TaqI G allele improves VDR mRNA stability and half-life, leading to an increased VDR synthesis and therefore directly altering vitamin D levels and consequently subsequent effects of vitamin D [55, 56].

A significant association was found between ApaI and infertility in the present meta-analysis. We observed a borderline and a significant protective association for the Aa vs. aa model (OR = 0.83, *P* = 0.05) and the dominant model (OR = 0.84, *P* < 0.05), however, no significant association was reported in other genetic model contrasts. These findings show that individuals who inherited ApaI SNP in a dominant form might be more protected against infertility. This polymorphism is in strong linkage disequilibrium with the poly(A) microsatellite located in the 3′ untranslated region [45] of the VDR gene, which appears to influence VDR messenger RNA stability and VDR translational activity (9). Sub-group analysis, however, showed a protective association against infertility in the PCOS subgroup under dominant (AA + Aa vs. aa), over-dominant, (Aa vs. AA + aa, AA vs. aa, and Aa vs. aa genetic models and a susceptibility association under the recessive genetic model in idiopathic infertility sub-group.

In our study, we noted a higher frequency of the genotype containing a mutated t allele of TaqI polymorphism. Interestingly, TaqI polymorphism was the only SNP that showed significant association with infertility overall and based on the etiology, excluding Over dominant genetic model. Our results showed that TaqI polymorphism may increase susceptibility to infertility under the allele contrast, recessive, dominant, homozygote, TT vs. Tt, Tt vs. tt genetic models. This indicates the If Taq t allele is a protective factor for infertility, then the infertility chance of patients with Taq t allele will be lower than that of patients with Taq T allele (OR > 1, *P* < 0.05). These data suggested the role of these genetic variants might be attributed with infertility due to the influence on the VDR function and consequently disturbed vitamin D metabolism or might be due to the LD with other VDR SNPs. These results suggest the special role of maternal setting genetic variants of the VDR gene in the etiology of this pregnancy complication. However, further research is required to determine what exactly FokI is acting as a marker for infertility.

As ~ 50% of patients with recurrent pregnancy loss (RPL) do not have a definite etiology, we further aimed to perform the meta-analysis of the association between VDR polymorphisms and recurrent miscarriage. The potential association of VDR polymorphisms with the etiology of recurrent miscarriages has been indicated in several studies [31, 45, 57]. Although with conflicting results, most of them suggested VDR SNPs association with RM in women. A study reported lower expression of VDR in trophoblastic, decidua, and serum villi in the RM group compared to the control, suggesting that impaired VDR expression in the first trimester of pregnancy may be associated with the occurrence of RM [48]. Accordingly, it could be suggested that VDR SNPs might be involved in the susceptibility and protection against RPL through influence on the VDR mRNA expression level and stability or due to the LD with other SNPs. In our study, we only found the association of FokI, and RPL in more than two studies, therefore the meta-analysis was performed for FokI polymorphism. Our data showed that FokI is significantly associated with a lower risk of RPL in allele contrast 9OR = 1.23, *P* = 0.034), recessive model (OR = 1.47, *P* = 0.010), and FF vs. Ff (OR = 1.46, OR = 0.016) genetic models. This indicates that carriers of FokI SNP might be more protected against RPL, however, it is required to be studied in larger sample sizes and to examine the exact functional effect of this SNP on the RPL etiology.

Because of racial differences, evidence of disease occurrence is not always accurate. This shows that various races have distinct effects on genetic background [58]. Therefore, based on subgroup analysis of different races, it can be found that the same polymorphisms in disease susceptibility in different populations play different roles. In our study, subgroup analysis suggested that the VDR gene BsmI polymorphism was significantly associated with susceptibility to infertility for the comparison of (AA vs. aa), (AA vs. Aa), and recessive model, and was protective SNP in the over-dominant genetic model in Asian population. For VDR gene TaqI polymorphism, it was significantly associated with susceptibility to infertility under the comparison of allele contrast (A vs. a), recessive model (AA vs. Aa + aa), dominant model (AA + Aa vs. aa), over-dominant (Aa vs. AA + aa), AA vs. aa, AA vs. Aa, Aa vs. aa genetic models in African and Asian population. However, for VDR gene ApaI polymorphism, it was protectively associated with infertility under dominant model (AA + Aa vs. aa), over-dominant (Aa vs. AA + aa), AA vs. aa, AA vs. Aa, Aa vs. aa genetic models and a susceptibility association was observed under recessive model (aa vs. Aa + AA) in Asian. FokI polymorphism was not significantly associated with infertility under any genetic models in any geographic population. The opposite association in different populations for an SNP in subgroup analysis might be due to ethnic differences. Of course, it also may be the difference in results caused by the insufficient number of studies included. We certainly need more and better research to get more reliable results.

Our study contains certain characteristics linked to study design that can help to strengthen the conclusions. The criteria for study selection were stringent, and such an exact selection guaranteed the right degree of analysis. Both groups (patients and controls) had participants who were similar in terms of age, ethnic background, and area of residence, reducing the impact of known confounders. In the genetic models, statistical power was adequate for genotype and allele frequencies of reported gene polymorphisms, as well as relationships between individual VDR polymorphisms and the probability of infertility/RPL. A drawback of this research is that we did not have original data, so we were unable to control for other factors such as circulating vitamin D levels, sun exposure, aspirin/NSAID use, stage disease, calcium, and vitamin D intake. The main drawback of the current study is the relatively small sample size and a lack of enough publications on the association of VDR SNPs and RPL. Finally, only four single nucleotide variations of the VDR gene were studied in this study, while, there are several more genetic variations that influence VD metabolism.

The limitation lies in the incapacity to explore diverse age groups through subgroup analyses, relying on the specified age range of 20 to 40 years in the primary studies. This constraint was underscored within the study's delineation of limitations regarding subgroup analyses grounded on age. Also, this study relied on secondary data sources, limiting our ability to control for potential confounding factors, including circulating vitamin D levels, sun exposure, aspirin/NSAID use, stage of disease, calcium, and vitamin D intake. The relatively small sample size in the current study reduced both statistical power and the generalizability of the results. Furthermore, insufficient publications on the association of VDR SNPs and RPL hindered comparison with previous studies. The examination in this study was confined to four single nucleotide variations of the VDR gene—FokI, BsmI, ApaI, and TaqI. However, numerous other genetic variations, such as CYP2R1, CYP27B1, CYP24A1, and GC, influence vitamin D metabolism. Consequently, our findings may not fully capture the genetic effects of vitamin D on RPL.

## Conclusion

Some comparisons revealed heterogeneity, but it was somewhat addressed by ethnicity-based subgroup analysis. According to our findings, VDR ApaI and FokI can have a role in infertility/recurrent miscarriage. These SNPs might be utilized to assess the risk of infertility/recurrent miscarriage. The observed relationships should be replicated in a bigger meta-analysis. Furthermore, expression studies are essential for fully comprehending the function of VDR polymorphisms in the etiology of infertility/recurrent miscarriage. Finally, investigations should be conducted to determine whether nutritional therapies such as vitamin D can provide a possible response to the hereditary propensity. Finally, our findings imply that VDR FokI and ApaI polymorphisms may be linked to infertility/recurrent miscarriage. However, more research with a larger sample size and considering other confounding factors is required in the future to reach a conclusive conclusion.

## Data Availability

The datasets used and/or analyzed during the current study are available from the corresponding author upon reasonable request.
